# Pharmacological Extracts and Molecules from *Virola* Species: Traditional Uses, Phytochemistry, and Biological Activity

**DOI:** 10.3390/molecules26040792

**Published:** 2021-02-03

**Authors:** María González-Rodríguez, Clara Ruiz-Fernández, Vera Francisco, Djedjiga Ait Eldjoudi, Yousof Ramadan Farrag AbdElHafez, Alfonso Cordero-Barreal, Jesus Pino, Francisca Lago, Manuel Campos-Toimil, Glaucimeire Rocha Carvalho, Thiago Melo Costa Pereira, Oreste Gualillo

**Affiliations:** 1 SERGAS (Servizo Galego de Saude) and IDIS (Instituto de Investigación Sanitaria de Santiago), The NEIRID Group (Neuroendocrine Interactions in Rheumatology and Inflammatory Diseases), Santiago University Clinical Hospital, Building C, Travesía da Choupana S/N, 15706 Santiago de Compostela, Spain; maria.gonzalez3112@gmail.com (M.G.-R.); clararf94@gmail.com (C.R.-F.); djidji.aiteldjoudi@gmail.com (D.A.E.); yousof.farrag@gmail.com (Y.R.F.A.); sitoalcorba@gmail.com (A.C-B.); jesus.pino.minguez@sergas.es (J.P.); 2 Molecular and Cellular Cardiology Group, SERGAS (Servizo Galego de Saude) and IDIS (Instituto de Investigación Sanitaria de Santiago), Research Laboratory 7, Santiago University Clinical Hospital, 15706 Santiago de Compostela, Spain; francisca.lago.paz@sergas.es; 3 Fisiología y Farmacología de las Enfermedades Crónicas (FIFAEC), Centro de Investigación en Medicina Molecular y Enfermedades Crónicas (CIMUS), Universidade de Santiago de Compostela, 15782 Santiago de Compostela, Spain; manuel.campos@usc.es; 4 Pharmaceutical Sciences Graduate Program, Vila Velha University (UVV), ES 29102-920 Vila Velha, Brazil; glauci_meire21@hotmail.com (G.R.C.); pereiratmc@gmail.com (T.M.C.P.); 5 Federal Institute of Education, Science and Technology (IFES), ES 29102-920 Vila Velha, Brazil

**Keywords:** Myristicaceae, pharmacology, phytotherapy, *Virola surinamensis*, *Virola sebifera*, resin, essential oil, flavonoid, lignan, alkaloid

## Abstract

*Virola* is the largest genus of Myristicaceae in America, comprising about 60 species of medium-sized trees geographically spread from Mexico to southern Brazil. The plant species of this genus have been widely used in folk medicine for the treatment of several ailments, such as rheumatic pain, bronchial asthma, tumors in the joints, intestinal worms, halitosis, ulcers, and multiple infections, due to their pharmacological activity. This review presents an updated and comprehensive summary of *Virola* species, particularly their ethnomedicinal uses, phytochemistry, and biological activity, to support the safe medicinal use of plant extracts and provide guidance for future research. The *Virola* spp.’s ethnopharmacology, including in the treatment of stomach pain and gastric ulcers, as well as antimicrobial and tryponosomicidal activities, is attributable to the presence of a myriad of phytoconstituents, such as flavonoids, tannins, phenolic acids, lignans, arylalkanones, and sitosterol. Hence, such species yield potential leads or molecular scaffolds for the development of new pharmaceutical formulations, encouraging the elucidation of not-yet-understood action mechanisms and ascertaining their safety for humans.

## 1. Introduction

Plant extracts have long been used as illness remedies in traditional medicine. Indeed, most of the new small-molecule and chemical entities of currently available drugs are derived from natural products, which are still widely used as sources of new drugs/leads [1]. Medicinal plants refer to any plants containing substances that can be used for therapeutic purposes or whose active principles can be used as precursors for the synthesis of new drugs. Tropical forest medicinal plants have been extensively used by indigenous peoples to treat several ailments, including cancerous, neurological, infectious, cardiovascular, immunological, inflammatory, and related diseases. Thus, the identification of possible bioactive compounds useful for medicine is of great importance to the pharmaceutical industry because they can provide new chemical entities or pharmacophores for drug development [2].

The *Virola* genus of the Myristicaceae family is found throughout tropical America, where these species have diverse local ethnobotanical uses [3]. Seed oils of *Virola* are used as machinery lubricants or soap ingredients or to make candles with intense light, little smoke, and fragrant smells. The seeds and arils can also be used to flavor beverages, such as chocolate drinks, while *Virola* wood is used for construction and carpentry [3]. Besides their non-medicinal benefits, *Virola* are perhaps best known for their hallucinogenic properties, which are often used in South American indigenous cultural practices [4]. Moreover, the reddish exudate of various *Virola* spp. is applied to treat skin conditions, alleviate tooth pain, and soothe colic, and as an anti-bleeding substance to treat ulcerating sores and wounds, as well as hemorrhoids. Additionally, the seed oils have many folk medicine applications, such as the treatment of asthma, rheumatism, tumors of the joints, intestinal worms, skin diseases, erysipelas, hemorrhoids, and bad breath [3]. Therefore, in the last few years, growing research has evaluated the biological effects of species from the *Virola* genus, identifying new promising bioactive compounds.

Here, we present an updated and comprehensive review on *Virola* species, focusing on their ethnopharmacological uses and phytochemistry. The botany of the *Virola* genus, in particular, the distinctive morphological and anatomic characteristics of various *Virola* species, are also described in order to support exact species identification, which is relevant for the establishment of unique and unequivocal pharmacological activities, and the reproducibility of research on the biological activity of their phytochemicals. The final aim of this review is to support the safe medicinal use of plant extracts and provide guidance for future research on new molecules with pharmaceutical activity.

## 2. Botany

### 2.1. Taxonomy 

The Myristicaceae family belongs to the Magnoliopsida class, Magnolianae superorder, and magnoliid order Magnoliales [5], and contains 21 genera and nearly 500 species of woody trees, shrubs, and some lianas. The *Virola Aubl*. genus is the fourth largest, and the biggest one in America, comprising around 60 species (please see [3] for a comprehensive taxonomic synopsis). An extensive description of all the *Virola* spp. is outside the scope of this review; instead, we summarize the available literature on the species with described ethnopharmacological uses and biological activity, namely, *Virola elongata*, *Virola peruviana*, *Virola venosa*, *Virola oleifera*, *Virola sebifera*, *Virola pavonis*, *and Virola surinamensis*.

### 2.2. Morphology and Anatomy

The plants of the *Virola* genus are characterized by distinctive growth forms and fruit. In particular, they grow as monopodial, orthotropic principal shoots, and plagiotropic lateral branches with distichous phyllotaxy—myristicaceous branching; the fruit’s maturation is accompanied by dehisce into two valves, displaying a single seed covered by a brightly colored laciniate aril rich in proteins, lipids, and sugars. These seeds are mainly dispersed by birds and monkeys [3]. Below, we mention the distinctive morphologic and anatomic characteristics of the main *Virola* species.

*Virola elongata* (synonym *Virola cuspidata* [6]) has an abaxial leaf surface sparsely pubescent, trichomes that are concolorous, trichome branches 0.1–0.2 mm long, peduncles of staminate inflorescences 0.07–0.15 cm thick, staminate flowers with the perianths 1.5–1.9 mm long, fruits 1.6–1.9 × 0.9–1.1 cm, a line of dehiscence canaliculate or smooth, and pericarp 0.5–0.7 mm thick [3].

*Virola peruviana* comprises trees up to 40 m, with tomentose and glabrescent branches, that have the following features: oblong leaves 15–35 × 6–11 cm; a cuspid or acuminate apex; a rounded or subchordinate base; a glabrous upper surface; a lower side with stellate trichomes; a sessile, glabrescent base; a glabrous upper surface; a sessile, glabrescent lower side with secondary stellate trichomes; a middle vein that is flat or slightly emergent and secondary ones flat or imprinted on the bundle, both emerging on the underside, secondary veins in 17–30 pairs; slightly brochidodromal camptodromes; inconspicuous tertiary venation [7]. 

*Virola pavonis* consists of trees up to 25 m with stilt roots, and ferruginous-tomentose or glabrate twigs, with leaves that are oblong–elliptical or obovate–elliptical. They have dimensions of 7–21 × 1.5–4.5 cm, a subacute or obtusely cuspid apex, an obtuse or attenuated base, a densely ferruginous-tumultuous upper surface, sessile trichomes, secondary veins imprinted on the upper surface emerging on the underside in 14–22 pairs in an arched brochidodrome, and petioles 4–13 mm long [7].

*Virola sebifera* are trees up to 30 m, sometimes with stilt, tabular roots; twigs that are tomentose and then puberulent and glabrescent; leaves that are ovate, oblong, elliptical, or oblong–deltoid; dimensions of 15–47 × 6–15 cm; a sharp or cuspid apex; a cordate base; a truncated, rounded, or broadly obtuse shape; an upper surface that is glabrous or sometimes pubescent in the middle vein; an underside that is dense and uniformly tomentose; persistent or evanescent trichomes that are dendritic, stellate, or irregularly branched; a middle vein that is slightly emergent and sometimes with two coasts and secondary ones imprinted on the upper surface, both emerging on the underside; secondary veins in 10–28 pairs, in a scalloped brochidodrome; and subparallel tertiary venation that is not very conspicuous [7].

*Virola venosa* are trees up to 30 m and have ellipsoidal or subglobular fruits, with dimensions of 19–22 mm long and 16–18 mm in diameter [8]. They have glabrous or sparse pubescent leaves on the bottom part and are wedged at the base, with thin venules and remarkable prominules in both parts, and inflorescences with 2–3 branches [9].

*Virola oleifera* is a semideciduous tree growing 20–30 m tall; the bole, which is usually straight and cylindrical, can be free of branches for up to 21 m, and it is 50–90 cm in diameter [10]. This species has oblong or linear leaves with parallel characteristic margins, about four times longer than wide, and powdery young lateral branches, which are few and short, with ovoid–ellipsoid fruits; it is acute or spat at the apex [9].

*Virola surinamensis* is an evergreen tree growing 25–35 m tall. The straight, cylindrical bole is buttressed; it can be free of branches for 15–18 m and is 60–90 cm in diameter. The leaf morphology implies numerous lateral veins, and stellate and sessile trichomes scattered on the abaxial surface of the leaf blades. This species has sometimes been identified as *Virola nobilis*. Both species’ leaves’ sizes and morphology may be similar; however, they tend to be smaller in *V. surinamensis*, and it also has inflorescence axes that are longer and with many more flowers. *V. surinamensis* is characterized by having a shorter perianth that is also fleshy to submembranous and smaller fruits that are also ovoid to subglobose, glabrescent, and with a thin pericarp [11]. 

### 2.3. Geographic Distribution

*Virola* species are extensively distributed in tropical America, being identified from Mexico to southern Brazil but rarely in El Salvador [3]. Of note, *V. surinamensis* is the only species found in the West Indies. The species richness is greater in western Amazonia, particularly in Brazil (~35 spp.), Colombia (~29 spp.), and Peru (~23 spp.). Central America, Costa Rica, and Panama have the highest concentration and diversity of *Virola* species. They are found mostly in humid forests below 800 m elevation. [3] Specifically, *V. elongata* grows in Panama to Brazil; *V. sebifera*, from Honduras to Brazil; both of them are very common in South America [3]. On the other hand, the species *V. pavonis* and *V. peruviana* are located in the Peruvian Amazon [12,13]. *V. venosa* is found in the Colombian Amazon region [14] and Brazil [15]. *V. oleifera* grows in the Atlantic Forest [16]; *V. sebifera*, in Honduras, Nicaragua, Costa Rica, and Panama [3]; and *V. surinamensis* is distributed from Costa Rica to the Amazon basin [17].

## 3. Ethnopharmacological Uses

Many of the *Virola* species are exploited for their medicinal properties by local inhabitants to treat and/or manage common human ailments, such as mental instability, infected wounds, skin infections, colic, and vitiligo [14]. Considering their relevant therapeutic activity in traditional practice, increasing research has been performed to support *Virola* species’ ethnopharmacological uses and identify their mechanisms of action as well as bioactive compounds (Table 1), ensuring toxicological safety.

*V. elongata* stem bark hydroethanolic extract is used for the treatment of stomach pain, indigestion, and gastric ulcers. This extract attenuates gastric ulceration by enhancing gastroprotection through its antioxidant properties and ability to significantly reduce gastric secretion and acidity in mouse and rat models of acute (acidified ethanol, piroxicam, and restraint-in-water stress) and chronic (acetic acid) gastric ulcers at doses of 100, 300, and 900 mg/kg p.o. (per os, orally) [6]. The aqueous infusion or the hydroethanolic macerate of the stem bark of *V. elongata* is also used in Brazilian and Ecuadorian indigenous folk medicine for the treatment of venereal diseases, abnormal vaginal secretions, infections, and cancer and for blood purification and wound healing [6]. Furthermore, *V. elongata* has shown tranquilizing effects and locomotor central nervous system depression, being used as a hallucinogenic drug because of its alkaloids [32].

**Figure 1:**
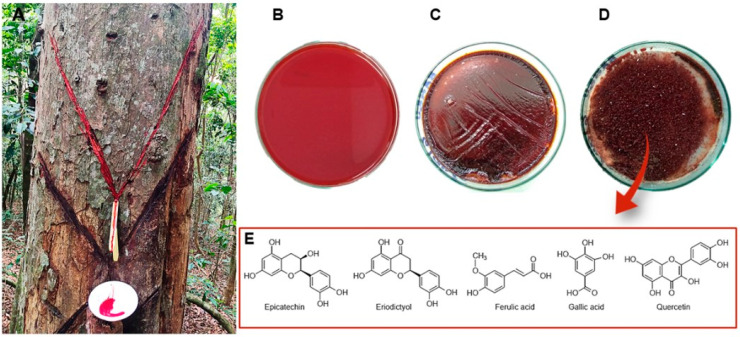
*Virola oleifera* in Espirito Santo, Brazil. The resin was collected from 0.5 cm-deep incisions in the tree trunk, as demonstrated in (**A**), and transferred into an amber glass vial (**B**). The fluid exudate was dried at 40 °C (**C**) and ground. Afterwards, the resin was sheltered from light at 4 °C until used as shown in (**D**). Previous chemical characterization by our research group revealed a high content of polyphenols, the main compounds demonstrated in (**E**) [16,33].

In popular medicine, *V. oleifera* is used to accelerate wound recovery and to treat pain and inflammatory conditions [9]. The oil extracted from the seeds of V. oleifera is popularly used for rheumatic pain, bronchial asthma, tumors in the joints, intestinal worms, halitosis, hemorrhoids, and skin diseases [9]. Validating its traditional use, *V. oleifera* resin (Figure 1) demonstrated gastroprotective effects (reduced gastric mucosal damage) in an ethanol/HCl and indomethacin ulcer-induction mouse model at 10 and 100 mg/kg when administered orally 30 min before gastric lesion induction, comparable to the reference control lansoprazole (3 mg/kg) [16]. Moreover, the chronic administration of antioxidant resin from *V. oleifera* (50 mg/kg administered orally) also attenuates atherogenesis in the atherosclerotic LDLr^−/−^ mouse model, through a decrease in vascular lipid deposition, the protection of the vascular endothelium and smooth muscle cells against peroxide-induced cytotoxicity, and the reduction of LPS-induced nitric oxide production in macrophages. Thus, *V. oleifera* resin’s anti-atherogenic activity might be mediated by systemic as well as local antioxidant and anti-inflammatory mechanisms [22]. Furthermore, the evaluation of the *V. oleifera* resin (300 mg/kg) in mice has suggested it to be a potential therapeutic solution for radiocontrast-induced nephropathy, due to its antioxidant and antiapoptotic effects and its ability to preserve kidney function by reducing renal dysfunction and morphological tubular injury [33]. Our current work also supports the ethnopharmacological use of *V. oleifera* in musculoskeletal malignancies, namely, multiple myeloma, and evidenced its potential as an adjuvant therapy to optimize dexamethasone’s pharmacologic effects and overcome cell drug resistance; nevertheless, herb–drug interactions with the proteasome inhibitor bortezomib could limit its clinical use (unpublished data).

*V. surinamensis* is widely used by the riverside inhabitants of the floodplain forest of the Mazagão River in the Brazilian Amazon to treat different disorders [34]. In particular, *V. surinamensis* seed oil is used topically for inflammatory skin conditions and as a preventive for microbial infections during wound healing. The oil is also used as a repellent. Leaf and bark decoctions are used as oral preparations for the treatment of inflammation in the digestive, urinary, and reproductive systems. Bark and fruit decoctions are used for intestinal infections, while exudate poultices are applied topically for furunculosis [34]. The resin obtained by cuts in the stem bark is also a reputed folk remedy in its natural form for the treatment of ulcers, gastritis, inflammation, cancer, erysipelas, colic, and dyspepsia [25,26]. Accordingly, the ethanolic extract (EE) of *V. surinamensis* resin has demonstrated preventive activity in rodent models of gastric mucosal disease [26]. Pre-treatment with the *V. surinamensis* resin EE (500mg/kg, p.o.) significantly inhibits mucosal injury (95% inhibition) in a gastric hemorrhagic ulcerative lesion model induced by an acidified ethanol solution. At the same dose, this extract also significantly reduced the formation of gastric lesions induced by indomethacin (39%), stress (45%), and pylorus ligature (31%) in mice, when compared to control animals. Interestingly, a comparison between the oral and intraduodenal administration of the *V. surinamensis* resin extract indicated therapeutic activity by local action, since no changes in gastric biochemical parameters (pH or total acid) were observed after intraduodenal administration [26]. Being the main constituent of *V. surinamensis* resin EE, epicatechin was suggested to be among the active principles responsible for the plant’s antiulcer activity [26]. An ethanolic extract of *V. surinamensis* stem bark was prepared and dried, and a 10% (*w*/*v*) solution diluted in dimethylsulfoxide was further tested for antimicrobial activity by measuring the diameter of the growth inhibition zone [25]. The extract exhibited antibacterial activity, particularly against Gram-negative microorganisms, thus providing preliminary scientific validation of its traditional medicinal uses. The phytoconstituents responsible for the antibacterial activity have not yet been identified, but it may be attributable to the presence of high concentrations of tannins, phenolic compounds, or catechin [25].

Furthermore, *V. pavonis* root and stem bark decoction is also known to be used for the treatment of skin infections and mucal mycosis [13].

## 4. Phytochemistry

Along with the determination of *Virola* spp.’s bioactivity, the characterization of the chemical composition, both compound identification and quantification, has also been performed. Furthermore, some studies have discussed the contribution of isolated compounds to the whole extract’s activity.

The chemical characterization of a stem bark hydroethanolic extract of *Virola elongata* revealed a content of 14.6% phenolic compounds (around 50% are flavonoids), among which are known antiulcer phenolic compounds, such as gallic acid, catechin, and rutin [6]. Quinic acid, 3,3′,4-trihydroxystilbene, juruenolid D, one catechin dimer, one C-glycosyl flavonoid, one polyketide, and two neolignans were also identified as major components of the stem bark hydroethanolic extract [6]. *V. elongata* (also known as V. cuspidata) contains the natural resveratrol analog (*Z*)-3,5,4′-trimethoxystilbene, which completely abrogated Caco-2 cell growth at 0.4 µM, while an 80% cell growth inhibition was observed at 0.3 µM (IC_50_: 0.25 µM). Analysis of its mechanisms of action indicated a blockage of the G2/M phase and the inhibition of tubulin’s polymerization, which plays a key role in neoplastic disease development [18]. Of note, under the same conditions, the (*E*)-3,5,4′-trihydroxystilbene (resveratrol) also showed a 70% inhibition of Caco-2 cell growth but at a higher concentration of 25 µM, which means that (*Z*)-3,5,4′-trihydroxystilbene is 100-fold more active than resveratrol [18]. The *β*-carbolines 6-methoxyharmalan and 6-methoxyharman, which have been suggested to possess some psychoactive effects, have also been isolated from *V. elongata* [19].

The isolation of 5-methoxy-*N,N*-dimethyltryptamine as well as the identification of *N,N*-dimethyltryptamine and 5-methoxytryptamine in *V. peruviana* has shown that the compounds may have hallucinogenic effects [12].

The bioassay-guided fractionation of a *V. sebifera* dichloromethane leaf extract isolated and elucidated the structure of polyketide 3,5-dihydro-2-(1′-oxo-3′-hexadecenil)-2-cyclohexen-1-one, which showed cytotoxic activity selective against the human tumor cell lines OVCAR03 (ovarian) and NCI-*ADR* (with a multidrug-resistance phenotype), in a dose-dependent way (IC_50_: 2–4 µg/mL) [20,21]. Additionally, the antiproliferative properties of *V. sebifera* (4′*Z*)1-hexadec-4′-enoyl-2,6-dihydroxybenzene [21] and polyketides from the leaves were reported, presenting moderate activity in all the cancer cell lines evaluated (IC_50_: 41–176 µg/mL) [20]. Additionally, two lignans were described in *V. sebifera*, rel-(8*R*, 8′*R*)—3 4:3′, 4′-bis-(methylendioxy)-7.7′-dioxo-lignan and (7′R, (‘S,8S)-2′-hydroxy-3,4:4,5′-bis-(methylenedioxy) -7oxo-2,7′-cyclolignan [35], whose antioxidant activity was further demonstrated, similarly to that for other natural aryltetralone lignans [36].

*V. venosa*’s blooms, fruits, and seeds contain flavonoids, lignans, arylalkanones, and sitosterol, while alkaloids and stilbenes are present in the leaves and roots [37]. Arylalkanones or acylarylrecorcinols have been found not only in *V. venosa* (both fruits and bark) but also in *V. elongata*, *V. surinamensis*, and *V. sebifera* [14]. Research investigating the α-glucosidase inhibitory activity of *V. venosa* and its bioactive compounds revealed that the methanolic extracts of the bark and leaf had a high content of phenolic compounds, demonstrating a high activity in antioxidant and α-glucosidase-inhibitory tests. These results are therapeutically relevant since inhibitors of α-glucosidase are potential compounds for the treatment of diabetes because they reduce diet-induced hyperglycemia by inhibiting this intestinal enzyme [15]. Furthermore, the activity-guided fractionation of *V venosa* methanolic bark and leaf extracts showed the presence of phenolic acids (ferulic acid, gallic acid, and *ρ*-coumaric acid) and flavonoids (quercetin, quercetrin, kaempferol, and catechin), and identified ferulic acid as the main bioactive compound for the antioxidant and α-glucosidase-inhibitory activities [15].

The renoprotective activity of *V. oleifera* resin may be attributable, at least in part, to the presence of antioxidant species, such as ferulic acid, gallic acid, and quercetin, previously identified by our group [33]. Moreover, we also described that the gastroprotective effect of this extract could be related to the content of polyphenols (~82%), namely, tannins (67.66 g/100 g of resin), phenolic acids, and flavonoids (48.257 ± 28.27 mg quercetin equivalent/100 g of resin), particularly epicatechin and eriodictyol [16]. *V. oleifera* leaf methanolic extracts have shown promising results as an analgesic treatment, which could be related to the oleiferin-C and flavonoid (quercitrin and astilbin) content [23]. The administration of *V. oleifera* leaf methanolic extract (10 mg/kg i.p.) inhibited acetic acid-induced abdominal constriction in mice similarly to aspirin or paracetamol [23]. Notably, oleiferin-C dose-dependently inhibited abdominal constriction by 76% at 29.2 µmol/kg (ID_50_: 17.2 µmol/kg), being 7.5-fold more active than aspirin (ID_50_: 125 µmol/kg) or paracetamol (ID_50_: 133 µmol/kg). In this study, the compounds quercitrin (quercetin-3-O-rhamnoside) and astilbin (taxifolin-3-O-rhamnoside) were also identified in the ethyl acetate fraction in a 2:1 ratio (quercitrin: astilbin), causing a reduction of 63% in abdominal muscle constriction. Since astilbin is inactive as an analgesic in mice, the effects of the quercitrin:astilbin mixture are likely due to quercitrin itself or to a potentiating synergistic effect between the two compounds [23].

Surinamensin is a natural neolignan isolated from the leaves of *V. surinamensis*, whose activity against *Leishmania donovani* amastigotes and promastigotes was tested in vitro based on its anti-schistosomal activity [27]. Steroids, lignans, flavonoids, and polyketides were also isolated from *V. surinamensis* [27]. The results revealed that surinamensin is active against *L. donovani* promastigotes but showed no selective toxicity when tested against the amastigotes in the mouse peritoneal macrophage model [28]. Besides, the 8.*O*.4′-neolignan 3,4,5-trimethoxy-8[3′,5′-dimethoxy-4′-propenylphenoxy]-phenylpropane was isolated from *Virola pavonis*, and it showed in vitro anti-*L. donovani* promastigote activity at 100 μM in an assay using extracellular promastigotes [28].

The neolignan grandisin was identified in *V. surinamensis* as well as in several Brazilian plant species used in popular medicine for the treatment of colic, inflammation, rheumatism, dyspepsia, and liver dysfunction. The antinociceptive and anti-inflammatory properties of grandisin have been investigated [24]. Treatment with grandisin (1, 3, and 10 mg/kg) dose-dependently reduced the number of acetic acid-induced abdominal writhes in mice, similarly to indomethacin at the highest dose used. Furthermore, the data indicated that the antinociceptive activity of grandisin is not dependent on motor incoordination or sedation due to the depressant effect in the CNS. The further evaluation of grandisin’s antinociceptive and anti-inflammatory activities in rodent models using the formalin test (a 60.5% reduction in paw licking time only in the inflammatory phase response), the croton oil-induced ear edema test (a 36.4% edema reduction at 10 mg/kg), and the carrageenan-induced peritonitis test (no effect at the concentrations tested) suggested that the mechanism of action of grandisin is not associated with the inhibition of events in the late stage of the inflammatory process, in which chemotaxis and cell migration occur. Instead, grandisin is hypothesized to reduce prostaglandin formation and/or activity through the inhibition of cyclooxygenase activity or prostaglandin receptor antagonist action. These results support the traditional use of grandisin-rich plants, such as *V. surinamensis*, in the treatment of symptoms caused by an inflammatory process such as pain and edema [24]. Grandisin was also identified as a potential drug candidate due to its antitumor and trypanocidal activities. Furthermore, grandisin is a competitive inhibitor of CYP2C9 and a competitive and mechanism-based inhibitor of CYP3A4/5 [31].

The essential oil obtained from adult and plantlet leaves of *V. surinamensis* has also been analyzed by GC–MS, and 11 monoterpenes, 11 sesquiterpenes, and three phenylpropanoids were identified. The plantlet essential oil caused 100% growth inhibition after 48 h in the development of the young trophozoite to the schizont stage, and the sesquiterpene nerolidol was identified as one of the active constituents, indicating a potential antimalarial use [29]. Dichioromethane extracts from *V. surinamensis* twigs showed in vitro trypanosomicidal activity against the trypomastigote form of *Trypanosoma cruzi* [30]. Costa et al. suggested that *V. surinamensis*’s antimicrobial activity is due to its tannin content [25].

## 5. Conclusions

Phytotherapy and the use of medicinal plants are traditionally part of the popular knowledge of different populations, users, and practitioners. The World Health Organization estimated that 80% of the developing countries’ population still relies on traditional medicines, medicinal plants being the most used, for its primary health care; they constitute an effective therapeutic form for the lower-income community. As evidenced in this review, *Virola* genus species are broadly used by local American inhabitants due to their beneficial effects on digestive conditions and rheumatic diseases, as well as inflammatory and infectious illnesses. In the last few years, several efforts have been made to validate the traditional uses of *Virola* spp. in human health and to identify bioactive phytochemicals. Several phenolic compounds, alkaloids, sesquiterpenes, lignans, and neolignans have been identified as antioxidant, anti-inflammatory, antinociceptive, and antimicrobial compounds. Of note, most of the studies were conducted on in vivo models, thus confirming their efficacy, therapeutic doses, and bioavailability. In this context, *Virola* genus species are evidenced as potential sources of therapeutic leads, offering novel opportunities in the search for new drugs. However, further basic and clinical research is needed to clarify and confirm the biological actions of *Virola* species and their bioactive molecules, minimizing the possible side and toxicological effects. Finally, studies on the potential herb–drug interactions as well as structure–activity relationships will be of relevance in the development of new pharmaceutics derived from *Virola* species phytochemicals.

## Figures and Tables

**Table 1:** Ethnomedicinal uses, biological activity, and bioactive phytochemicals of *Virola* species.

Species	Plant Part Used	Type of Extract	Traditional Uses	Bioactivity and Mechanism of Action (In Vitro and In Vivo Models)	Type of Compound	Compound Identified	Ref.
***V. elongata*** **(or** ***V. cuspidata*** **)**	Stem bark	Hydro- ethanolic	Stomach pain Indigestion Gastric ulcers	Gastroprotective/antiulcer: antioxidant, gastric secretion, and total acidity reduction (rodent models of acute and chronic gastric ulcers)	Phenolic acids	Gallic acid	[6]
					Stilbenes	3,3′,4-trihydroxystilbene	
					Flavonoids	Catechin Rutin	
					Neolignans	Arylnaphtalene Dibenzylbutane	
				Antiproliferative in colon adenocarcinoma cells: cell cycle arrest in G2/M, anti-mitotic activity through tubulin polymerization inhibition (Caco-2 cell line)	Stilbenes	(*Z*)-3,5,4′-trihydroxystilbene (resveratrol analog)	[18]
				Psychoactive	Alkaloids (β-carbolines)	6-methoxyharmalan 6-methoxyharman	[19]
***V. peruviana***	Coarse bark	Petroleum ether extraction	Hallucinogenic		Alkaloids	5-methoxy-*N,N*-dimethyltryptamine *N,N*-dimethyltryptamine 5-methoxytryptamine	[12]
***V. sebifera***	Leaves	Dichloromethane		Cytotoxic and antiproliferative in cancer cells (human ovarian OVCAR03 and multidrug-resistance-phenotype NCI-ADR tumor cell lines)	Polyketide	3,5-dihydro-2-(1′-oxo-3′-hexadecenil)-2-cyclohexen-1-one	[20,21]
					Acylresorcinol	(4′*Z*)1-hexadec-4′-enoyl-2,6-dihydroxybenzene	
***V. oleifera***	Seed	Oil	Rheumatic pain Bronchial asthma Joint tumors Intestinal worms Halitosis Hemorrhoids Skin diseases				[9]
	Resin	Plant fluid exudate collection	Chronic wound healing Hemoptysis Leukorrhea Diarrhea	Gastroprotective/antiulcer: antioxidant activity (ethanol/HCl and indomethacin ulcer-induction mouse models)	Flavonoids	Epicatechin Eriodictyol	[16]
					Tannins		
				Atheroprotective: systemic and local antioxidant and anti-inflammatory activity (LDLr^−/−^ mouse model)			
				Nephroprotective: antioxidant and antiapoptotic in renal glomerular and tubular cells (contrast-induced nephropathy mouse model)	Phenolic acids	Ferulic acid Gallic acid	[22]
					Flavonoids	Quercetin	
	Leaves	Methanolic	Analgesic	Analgesic (writhing test in mice)	Flavonoids	Quercitrin Astilbin	[23]
					Neolignans	Oleiferin-C	
***V. surinamensis***	Resin	Ethanolic	Ulcer Gastritis Inflammation Cancer	Antiulcer: inhibition of gastric lesions (ethanol/HCl, indomethacin, stress, and pylorus ligature ulcer-induction mouse models)	Flavonoids	Epicatechin	[24,25,26]
	Stem bark	Ethanolic	Inflammation Cancer	Antibacterial Antifungal (diameter of the inhibition zone test)	Phenolic compounds		
					Flavonoids	Catechin	
					Tannins		
	Leaves	Infusion	Colic Dyspepsia	Antinociceptive (acetic acid-induced abdominal writhing test and formalin test in mice) Anti-inflammatory (croton oil-induced ear oedema test in mice)	Neolignans	Grandisin	
		Hexane		Anti-schistosomal and anti-leishmanial activity (in vitro tests)	Neolignans	Surinamensin	[27,28]
	Plantlet leaves	Essential oil	Malaria	Antimalarial (in vitro test)	Sesquiterpenes	Nerolidol	[29]
	Twig	Dichloromethane		Trypanosomicidal activity (in vitro test against *Trypanosoma cruzi*)	Lignans and neolignans	Veraguensin Grandisin	[30]
				Inhibition of CYP2C9 and CYP3A4/5	Neolignans	Grandisin	[31]
***V. venosa***	Bark Leaves	Methanolic	Antioxidant	Antioxidant and α-glucosidase-inhibitory activity (DPPH and α-glucosidase-inhibition assay)	Phenolic acids	Ferulic acid Gallic acid ρ-coumaric acid	[15]
					Flavonoids	Quercetin Quercitrin Kaempferol Catechin	
					Neolignans	Oleiferin-C	
***V. pavonis***	Root Bark stem	Decoction	Skin infection Oral mycosis	Antimicrobial (in vitro tests)			[13]

## Data Availability

The data presented in this study are available in the cited articles.

## References

[1] Newman D.J., Cragg G.M. (2020). Natural products as sources of new drugs over the nearly four decades from 01/1981 to 09/2019. J. Nat. Prod..

[2] Zimmermann T., Drašar P.B., Rimpelová S., Christensen S.B., Khripach V.A., Jurášek M. (2020). Large Scale Conversion of Trilobolide into the Payload of Mipsagargin: 8-*O*-(12-Aminododecanoyl)-8-*O*-Debutanoylthapsigargin. Biomolecules.

[3] Santamaría-Aguilar D., Aguilar R., Lagomarsino L.P. (2019). A taxonomic synopsis of Virola (Myristicaceae) in Mesoamerica, including six new species. PhytoKeys.

[4] McKenna D.J., Towers G., Abbott F. (1984). Monoamine oxidase inhibitors in South American hallucinogenic plants: Tryptamine and β-carboline constituents of Ayahuasca. J. Ethnopharmacol..

[5] (2019). ITIS Standard Report Page: Lycopodiophytina. https://www.itis.gov/servlet/SingleRpt/SingleRpt?search_topic=TSN&search_value=18122#null).

[6] De Almeida G.V.B., Arunachalam K., Balogun S.O., Pavan E., Ascêncio S.D., Soares I.M., Zanatta A.C., Vilegas W., Macho A., Martins D.T.D.O. (2019). Chemical characterization and evaluation of gastric antiulcer properties of the hydroethanolic extract of the stem bark of Virola elongata (Benth.) Warb. J. Ethnopharmacol..

[7] Ureta Adrianzén M. (2010). Taxonomic Review of the Myristicaceae Family from Central Forest, Oxapampa-Perú. Intropica.

[8] Plantes et Botanique. https://archive.is/20070519184701/http://www.plantes-botanique.be/e2-Myristicaceae-Virola-venosa.

[9] Rodrigues W.A. (1980). Revisão taxonômica das espécies de Virola Aublet (Myristicaceae) do Brasil. Acta Amaz..

[10] Tropical Plants Database, Ken Fern. http://tropical.theferns.info/viewtropical.php?id=Virola+oleifera.

[11] Tropical Plants Database, Ken Fern. http://tropical.theferns.info/viewtropical.php?id=Virola+surinamensis.

[12] Lai A., Tin-Wa M., Mika E.S., Persinos G.J., Farnsworth N.R. (1973). Phytochemical Investigation of Virola peruviana, A New Hallucinogenic Plant. J. Pharm. Sci..

[13] Roumy V., Macedo J.C.R., Bonneau N., Samaillie J., Azaroual N., Encinas L.A., Rivière C., Hennebelle T., Sahpaz S., Antherieu S. (2020). Plant therapy in the Peruvian Amazon (Loreto) in case of infectious diseases and its antimicrobial evaluation. J. Ethnopharmacol..

[14] Castro E., Suarez L.E.C., Siengalewicz P., Gutmann R., Czermak G., Brueggeller P. (2004). 3,6-Dihydroxy-2-(11-phenylundecanoyl)cyclohex-2-en-1-one from Virola venosa bark. Acta Crystallogr. Sect. C Cryst. Struct. Commun..

[15] Fernandes K.R.P., Bittercourt P.S., De Souza A.Q.L., De Souza A.Q.L., Da Silva F.M.A., Lima E.S., Acho L.D.R., Nunomura R.D.C.S., Teixeira A.F., Koolen H.H.F. (2019). Phenolic compounds from Virola venosa (Myristicaceae) and evaluation of their antioxidant and enzyme inhibition potential. Acta Amaz..

[16] Pereira A.C.H., Lenz D., Nogueira B.V., Scherer R., Andrade T.U., Da Costa H.B., Romão W., Pereira T.M.C., Endringer D.C. (2016). Gastroprotective activity of the resin from Virola oleifera. Pharm. Biol..

[17] Riba-Hernández P., Segura J.L., Muñoz-Valverde J. (2016). Female fruit production depends on female flower production and crown size rather than male density in a continuous population of a tropical dioecious tree (Virola surinamensis). Am. J. Bot..

[18] Chabert P., Fougerousse A., Brouillard R. (2006). Anti-mitotic properties of resveratrol analog (Z)-3,5,4′-trimethoxystilbene. BioFactors.

[19] Cassady J.M., E Blair G., Raffauf R.F., E Tyler V. (1971). The isolation of 6-methoxyharmalan and 6-methoxyharman from Virola cuspidata. Lloydia.

[20] Denny C., Zacharias M.E., Lúcia A., Gois T., Amaral C.E., Bittrich V., Kohn L.K., De Oliveira I.M., Alexandre R., Rodrigues F. (2008). Antiproliferative Properties of Polyketides Isolated from Virola Sebifera Leaves. Phytother. Res..

[21] Pagnocca F.C., Ribeiro S.B., Torkomian V.L.V., Hebling M.J.A., Bueno O.C., Da Silva O.A., Fernandes J.B., Vieira P.C., Silva M.F.D.G.F.D., Ferreira A.G. (1996). Toxicity of lignans to symbiotic fungus of leaf-cutting ants. J. Chem. Ecol..

[22] Coutinho P.N., Pereira B.P., Pereira A.C.H., Porto M.L., De Assis A.L.E.M., Destefani A.C., Meyrelles S.S., Vasquez E.C., Nogueira B.V., De Andrade T.U. (2017). Chronic administration of antioxidant resin from Virola oleifera attenuates atherogenesis in LDLr -/- mice. J. Ethnopharmacol..

[23] Kuroshima K.N., De Campos F., De Souza M.M., Yunes R.A., Monache F.D., Filho V.C. (2001). Phytochemical and Pharmacological Investigations of Virola oleifera Leaves. Z. Nat. C.

[24] Carvalho A.A.V., Galdino P.M., Nascimento M.V.M., Kato M.J., Valadares M.C., Cunha L.C., Costa E.A. (2010). Antinociceptive and antiinflammatory activities of grandisin extracted fromVirola surinamensis. Phytother. Res..

[25] Costa E.S., Hiruma-Lima C.A., Lima E.O., Sucupira G.C., Bertolin A.O., Lolis S.F., Andrade F.D.P., Vilegas W., Souza-Brito A.R.M. (2008). Antimicrobial activity of some medicinal plants of the Cerrado, Brazil. Phytother. Res..

[26] Hiruma-Lima C.A., Batista L.M., De Almeida A.B.A., Magri L.D.P., Dos Santos L.C., Vilegas W., Brito A.R.M.S. (2009). Antiulcerogenic action of ethanolic extract of the resin from Virola surinamensis Warb. (Myristicaceae). J. Ethnopharmacol..

[27] Barata L.E., Baker P.M., Gottlieb O.R., Rúveda E.A. (1978). Neolignans of Virola surinamensis. Phytochemistry.

[28] Barata L.E.S., Santos L.S., Ferri P.H., Phillipson J., Paine A., Croft S.L. (2000). Anti-leishmanial activity of neolignans from Virola species and synthetic analogues. Phytochemistry.

[29] Lopes N.P., Kato M.J., Eloısa H.D.A., Maia J.G., Yoshida M., Planchart A.R., Katzin A.M. (1999). Antimalarial use of volatile oil from leaves of Virola surinamensis (Rol.) Warb. by Waiãpi Amazon Indians. J. Ethnopharmacol..

[30] Lopes N.P., Chicaro P., Kato M.J., De Albuquerque S., Yoshida M. (1998). Flavonoids and Lignans fromVirola surinamensisTwigs and theirin vitroActivity againstTrypanosoma cruzi. Planta Medica.

[31] Habenschus M., Moreira F.D.L., Lopes N.P., De Oliveira A.R.M. (2017). In Vitro Inhibition of Human CYP450s 1A2, 2C9, 3A4/5, 2D6 and 2E1 by Grandisin. Planta Medica.

[32] Macre W.D., Towers G.N. (1984). An ethnopharmacological examination ofVirola elongata bark: A South American arrow poison. J. Ethnopharmacol..

[33] Bôa I.S.F., Porto M.L., Pereira A.C.H., Ramos J.P.L., Scherer R., Oliveira J.P., Nogueira B.V., Meyrelles S.S., Vasquez E.C., Endringer D.C. (2015). Resin from Virola oleifera Protects Against Radiocontrast-Induced Nephropathy in Mice. PLOS ONE.

[34] Sarquis R.D.S.F.R., Sarquis Í.R., Sarquis I.R., Fernandes C.P., Da Silva G.A., E Silva R.B.L., Jardim M.A.G., Sánchez-Ortíz B.L., Carvalho J.C.T. (2019). The Use of Medicinal Plants in the Riverside Community of the Mazagão River in the Brazilian Amazon, Amapá, Brazil: Ethnobotanical and Ethnopharmacological Studies. Evid. Based Complement. Altern. Med..

[35] Rezende K.R., Kato M.J. (2002). Dibenzylbutane and aryltetralone lignans from seeds of Virola sebifera. Phytochemistry.

[36] Rezende K.R., Davino S.C., Barros S.B., Kato M.J. (2005). Antioxidant activity of aryltetralone lignans and derivatives fromVirola sebifera(Aubl.). Nat. Prod. Res..

[37] Kato M.J., Yoshida M., Gottlieb O.R. (1992). Flavones and lignans in flowers, fruits and seedlings ofVirola venosa. Phytochemistry.

